# Unraveling the genetic and molecular bases of heterosis in a TGMS-based two-line rice hybrid derived F_2_ segregating population

**DOI:** 10.3389/fpls.2025.1722476

**Published:** 2026-01-16

**Authors:** Faraz Azeem, Jauhar Ali, Tonette. P. Laude, Seyed Mahdi Hosseiniyan Khatibi, Varunseelan Murugaiyan, Neeraj Kumar, Angelito Galang, Madonna Dela Paz, Erik Jon De Asis, Atul Singh, Christian John Robiso, Carla Francesca Besa, Pompe C. Sta. Cruz, Eureka Teressa M. Ocampo, Jose E. Hernandez

**Affiliations:** 1 Rice Breeding Innovation Department, International Rice Research Institute, Los Baños, Philippines; 2 Institute of Crop Science, College of Agriculture and Food Science, University of the Philippines Los Baños, Los Baños, Philippines; 3 Department of Genetics and Plant Breeding, Indira Gandhi Krishi Vishwavidyalaya, Raipur, Chhattisgarh, India

**Keywords:** heterosis, QTL mapping, candidate genes, gene expression, F_2_ segregating population, thermosensitive genic male sterility (TGMS), two-line hybrid rice, yield-related traits

## Abstract

**Introduction:**

Heterosis has played a pivotal role in enhancing rice yield, yet its genetic and molecular bases remain only partially understood, particularly in two-line thermosensitive genic male sterile (TGMS) hybrid systems.

**Method:**

In this study, we dissected the genetic architecture underlying heterosis using an F₂ segregating population comprising 392 individuals, derived from the elite TGMS-based hybrid IR144693H (IRAC-43S×IRV932). High-density genotyping was performed using the 1k-Rice Custom Amplicon (1k-RiCA) SNP panel, coupled with comprehensive phenotyping of yield and yield-related traits under field conditions.

**Results:**

Quantitative trait locus (QTL) analysis identified 24 main-effect QTLs associated with seven agronomic and physiological traits, explaining 4.1% to 67.5% of the phenotypic variance. Major-effect QTLs were detected for number of tillers (qNT3011 and qNT6011 on chromosome 11), unfilled grains (qUFG7.2 and qUFG9), and thousand grain weight (qTGW12.1), highlighting key genomic regions contributing to heterosis. The F₂ segregating population exhibited extensive phenotypic variation and transgressive segregation for multiple traits, underscoring the complex and polygenic nature of heterosis in TGMS-based rice hybrid systems.

**Discussion:**

In the absence of system-specific reference genomes and transcriptomic resources for two-line rice hybrids, candidate gene identification was conducted using the Nipponbare reference genome and publicly available expression datasets. Several biologically relevant genes were prioritized within major QTL intervals, including SPP (sucrose-phosphate phosphatase), GW2 (grain width regulator), DEP1 (panicle architecture), and OsCCaMK (calcium/calmodulin-dependent protein kinase), supported by positional evidence and tissue-specific expression patterns.Overall, this study provides a high-resolution dissection of heterosis-associated genomic regions in a TGMS-derived F₂ segregating population and delivers valuable candidate loci for marker-assisted selection and functional validation. These findings advance our understanding of heterosis in two-line hybrid rice and offer practical insights for the development of next-generation high-yielding hybrids.

## 1 Introduction

Rice (*Oryza sativa* L.) is a primary food source for approximately half of the global population, mainly in Asia. With the world population projected to reach 10 billion by 2050, the demand for rice will continue to increase, requiring an annual increase of at least 100 million metric tons to maintain food security (Seck et al., 2012; Merrey et al., 2018). In 2018, 167.1 million hectares produced 782 million tons of rice, yielding a global average of 4.68 tons per hectare (Kesh et al., 2023). Nevertheless, production must increase to keep pace with the growing global food demand in the coming decades (Ali et al., 2021). Achieving future yield goals is becoming increasingly challenging due to shrinking land, rising input costs, water scarcity, and climate-related challenges, underscoring the need for stable and sustainable rice production. Future rice varieties must increase yield, have better nutritional value, withstand environmental stresses, and have a lower carbon footprint (Wing et al., 2018). By providing a 15%–20% yield advantage over inbred varieties, hybrid rice technology underscores the goal for future rice varieties to enhance yield, nutritional quality, stress tolerance, and sustainability (Cheng et al., 2007; Li et al., 2007b; Krieger et al., 2010). Hybrid rice primarily employs three-line (CMS-based) and two-line (TGMS or PGMS)-based breeding technologies and is currently being cultivated on over 25 million hectares globally. Notably, the two-line hybrid rice breeding approach is expanding rapidly, with the level of heterosis exceeding 25%–30% over elite hybrid and inbred checks. Therefore, it raises fundamental questions about the genetic basis of heterosis and how it can be manipulated to achieve higher grain yields (Huang et al., 2016).

The temperature-sensitive genic male sterility (TGMS) system, based on the recessive *tms5 gene*, induces male sterility at higher mean temperatures (>26°C) and is fertile at lower mean temperatures (<24°C) (Zhou et al., 1988; Zongxiu et al., 1989). Male pollen sterility is induced between stage II and stage IV of panicle initiation of the TGMS rice plant if it receives a higher mean temperature (>26°C), allowing hybrid rice seed production. This fertility–sterility alteration of TGMS lines enables the robust production of hybrid rice seeds and the self-seeding multiplication of TGMS lines (Ali, 1993). TGMS sources have either an induced or spontaneous origin, like Annong S-1 and Anxiang S (Zhouci et al., 1990; Lu et al., 1994) in China and Norin PL 12 (Maruyama et al., 1990, 1991) and SA2 in India (Ali, 1993; Siddiq and Ali, 1999; Hussain et al., 2012). Compared to the three-line method, two-line hybrid rice technology offers several advantages. These include wider genetic diversity germplasm as pollen parents and simplified hybrid seed production, which provides opportunities to profit from higher heterosis and easier breeding and hybrid seed production procedures (Chen et al., 2020; Ali et al., 2021). This approach is crucial for boosting agricultural production to meet global food demand amid a rapidly increasing population and climate change (Wang, 2015) The TGMS system is highly sensitive to temperature, with even slight fluctuations within the range of 22°C–24°C potentially leading to significant impacts on the seed set due to sterility (Xiaohe et al., 1992; Zhou et al., 2016). The *tms5* gene is crucial in regulating thermosensitive sterility in many TGMS lines despite its ability to alter sterility-inducing temperatures across different genetic backgrounds. Two sterile lines were initially produced using the temperature-sensitive sterile gene *tms5* (Borkakati and Virmani, 1996; Reddy et al., 2000; Yu et al., 2017). The F_2_ segregating population from the elite crosses (F_1_) is one of the potential choices for dissecting the genetic basis of heterosis because three genotypes are present with a ratio of 1:2:1 for a single locus with two alleles. The equal distribution of allelic combinations between different loci allows for a precise estimate of the genetic effect. Genotyping F_2_ individuals is more feasible and relatively inexpensive, offering a broad phenotypic variation among the population and allowing us to understand transgressive segregation for yield traits. The F_2_ segregation population showed a pollen fertility ratio of 15 fertile to 1 sterile (Zhang et al., 1994).

Many male sterile lines with an *indica* background exhibited similar segregation genetic ratios, similar to Peiai64S (Subudhi et al., 1997; Dong et al., 2000; Zhou et al., 2011). Nevertheless, the successful implementation of this innovative male sterility technique depends on understanding the fertility behavior of TGMS lines (Chandirakala et al., 2008). Additionally, a large-scale study has demonstrated the potential of F_2_ mapping populations in hybrid rice. For example, in a previous report (Huang et al., 2016), a population of 10,074 F_2_ lines was exploited to identify key heterosis-related loci in hybrid rice (Huang et al., 2016). Recently, 2,839 cultivars and 9,839 F_2_ populations were analyzed, showing that *indica–indica* hybrid breeding expands the genetic resources, pyramiding favorable alleles, and eliminates deleterious alleles through combinatorial selection (Yuan, 1990; Mengliang et al., 1999; Chuan-gen et al., 2007; Gu et al., 2023). QTL mapping in F_2_ populations has become a critical tool to understand genetic and molecular mechanisms of heterosis (Wu et al., 2022; Xie et al., 2022; Zhou et al., 2024). Integrating high-density SNP genotyping with trait phenotyping would allow us to understand QTLs controlling yield and its components, which would enable us to pinpoint possible candidate loci for molecular breeding. The 1k-Rice Custom Amplicon (1k-RiCA) SNP platform is particularly suited for high-resolution mapping in rice (Arbelaez et al., 2019). In this study, we investigated an F_2_ population derived from the TGMS-based high-yielding rice hybrid IR144693H, a cross between IRAC-43 × IRV932, to detect QTLs linked with grain yield and its component traits. Primarily, we aimed to identify key genomic regions and candidate genes contributing to heterosis and provide a foundation for marker-assisted selection (MAS) and genomic-assisted breeding of next-generation TGMS hybrids.

## 2 Materials and methods

An F_2_ segregating population of the high-yielding TGMS-based hybrid IR144693H (IRAC43S ^×^ IRV932) was used to map in this study. A total of 2,000 F_2_ plants were established, and 392 plants (genotype) were randomly selected. The female parent IRAC-43 is a high general combining ability (GCA) TGMS line, while IRV932 is a stress-tolerant pollen parent. Freshly harvested seeds were used for sowing after breaking seed dormancy at 50°C for 3 days. Seeds of the F_2_ and parental lines were germinated (150 cm × 90 cm × 15 cm) in metal trays filled with sterilized clay loam soil in a greenhouse. Twenty-one-day-old seedlings were then transplanted to Tublay, Benguet [16.516772°N latitude, 120.638592°E longitude, and an altitude of 3,517 feet (1,072 m) above mean sea level]. Tublay experiences a low mean temperature (~24°C), ensuring fertility expression in TGMS lines. The experiment was laid out with a 20 cm × 20 cm spacing, totaling 80 rows by 25 hills (76 m² plot). For the phenotyping and genotyping, each plant was labeled using the Enterprise Breeding System (EBS). Environmental condition was recorded using an EM50 datalogger (Decagon Devices, Pullman, WA, United States). A fertilizer rate of 120–30–30–5 kg NPK-Zn ha^−1^ was applied during the 2022 wet season at Tublay.

### 2.1 Phenotyping for yield and its component traits

Phenotypic evaluation was conducted during the 2022 wet season, in accordance with the IRRI Standard Evaluation System (SES). A total of 28 traits were measured at 30, 60, and 90 days after sowing (DAS), including plant height (PH), soil plant analysis development (SPAD), normalized difference vegetation index (NDVI), stomatal conductance (gs), days to 50% flowering (DFF), number of tillers (NT), flag leaf length (FLL), flag leaf width (FLW), panicle length (PL), productive tillers (PT), grain yield per plant (YPP), panicle dry weight (PDW), leaf dry weight (LDW), filled grains (FG), unfilled grains (UFG), grain length (GL), grain width (GW), thousand grain weight (TGW), and biomass (BM).

SPAD values were measured using a SPAD-502 plus chlorophyll meter (Konica Minolta, Japan) by recording readings from the leaf tip, middle, and base of the fully expanded leaf. NDVI was measured using a GreenSeeker handheld optical sensor (Trimble Inc., USA) positioned 0.8–1.0 m above the canopy, maintaining a constant height and orientation. Stomatal conductance (gs, mmol m^−2^ s^−1^) was measured using the AP4 porometer (Delta-T Devices, Cambridge, UK), at the booting and grain filling stages in the middle of the flag leaf under clear environmental conditions between 09:00 a.m. and 12:30 p.m., minimizing diurnal variation. All instruments were calibrated before each measurement session to ensure accuracy and consistency. For convenience, stage-specific values are denoted as SCBS (gs at the booting stage) and SCGF (gs at the grain filling stage) throughout the tables and figures. For each physiological trait, three measurements per plant were recorded.

DFF was recorded as the number of days from sowing to when 50% of the F_2_ plants in the field begin flowering. Before harvest, NT, FW (cm), FLL (cm), PL (cm), and PT were recorded. Each F_2_ plant was harvested separately, and YPP (cm) was weighed at 14% moisture content. For the primary panicle, FG and UFG were counted. GL (mm), GW (mm), TGW (g), and PDW (g) were measured for each F_2_ plant. Plants were oven-dried at 70°C for 5 days, after which BM (g) was measured. The mean values for all traits were used for downstream analysis.

### 2.2 Leaf sample collection and genotyping

Young leaf tissues from 394 F_2_ population and their parents were collected in labeled zip bags for DNA extraction. Genomic DNA was extracted using the CTAB-based protocol (Dilla-Ermita et al., 2017). DNA quality was verified on 1% agarose gel, and the concentration was determined using PicoGreen^®^ (https://www.biotek.com) and a Qubit 2.0 fluorometer. The DNA concentration was adjusted to ~10 ng/μL for library preparation. Utilizing 1k-RiCA, genome sequencing was performed for 394 individuals (Arbelaez et al., 2019) at AgriPlex IRRI Genomics. SNP data were integrated with physical position and chromosome number in HapMap format, and markers were filtered for polymorphism between parents.

### 2.3 Statistical analyses

Using the STA extension in QTL IciMapping v4.2, basic statistics of phenotypic data for yield and its component traits were calculated among the 392 F_2_ individuals (Meng et al., 2015). The violin plot for traits was plotted using “R” (R version 3.6.1). Pairwise Pearson correlation analysis was carried out among the heterosis-related traits, using a two-tailed *P*-value with two significant levels (*P* = 0.05 and 0.01), and a heatmap was generated using the “*pheatmap*” package in RStudio. Principal component analysis (PCA) was carried out to observe the pattern of variation among the 392 rice genotypes and the relationship among the lines and their traits using the “*FactoMineR*” package in RStudio. Based on the correlation analysis, highly correlated traits were identified, and frequency distribution histograms were generated for key physiological and morphological traits to characterize phenotypic variation within the F_2_ segregating population.

### 2.4 QTL mapping

From 1,008 SNPs, a total of 782 polymorphic markers were retained after filtering. Alleles were coded as follows: AA = pollen parent (IRV932), BB = TGMS female parent (IRAC-43), and heterozygotes as “HH”. For genetic map construction (Li et al., 2007a; Meng et al., 2015), Kosambi’s mapping function was used to convert recombination frequencies to centimorgan (cM) distances. QTL analysis was performed using the BIP module under interval mapping (IM). The LOD threshold was obtained based on the permutation test (1,000 permutations at *P* < 0.005) for each trait (Doerge and Churchill, 1996). The total phenotypic variance explained (PVE) by each QTL was estimated by 1 − 10^−2LOD/^*^n^*, where *n* represents the sample size and LOD is the LOD score. The confidence interval of QTLs was delimited by the flanking marker within a 1 − LOD drop from the estimated QTL position. After identifying QTLs on the genetic linkage map, the corresponding flanking SNP markers were aligned to the physical genome based on their chromosomal positions, allowing each QTL to be accurately anchored and represented as a defined physical interval. The QTL detected for each trait was named using the trait attribute, followed by the number representing the chromosome location and the numerical order of the identified QTL on the chromosome. For instance, qTGW12.2 denotes the second QTL identified for thousand grain weight on chromosome 12 (Pang et al., 2017). The Kruskal–Wallis test evaluated allelic effects on traits (Kruskal and Wallis, 1952). This approach enhanced QTL detection accuracy by controlling background variation and minimizing type I errors.

### 2.5 Candidate gene identification and tissue-specific expression

Main-effect QTL regions associated with yield and yield component traits were mined for candidate genes. These candidate genes were identified using significant SNP markers, based on data from the MSU Rice Genome Annotation Project (http://rice.uga.edu/, https://ricepilaf.irri.org/) (**Supplementary Table S3**). In the absence of high-quality reference genomes and system-specific transcriptome resources for two-line hybrid rice systems, candidate gene discovery was conducted using the *Oryza sativa* ssp. *japonica* “Nipponbare” reference genome and publicly available expression datasets. All QTL intervals were aligned to the Nipponbare assembly, and gene models within each interval were extracted, with peak SNPs showing the strongest statistical association used to identify the nearest or overlapping gene models, which were designated as the primary candidate loci. To assess their functional relevance, tissue-specific expression patterns of these genes were examined using publicly available high-resolution RNA-seq data encompassing major vegetative and reproductive tissues (http://rice.uga.edu/, https://ricepilaf.irri.org/) (**Supplementary Table S2**). Expression enrichment in tissues, consistent with the biology of the mapped trait, was used as a key criterion for prioritization. Functional annotations, gene ontology information, and supporting literature were then integrated to refine the list, resulting in a set of candidate genes supported by both positional evidence and stage-specific expression profiles for downstream validation.

## 3 Results

### 3.1 Distribution of agronomic and physiological traits

Descriptive statistics and frequency distribution of agronomic and physiological traits showed substantial phenotypic variation among the F_2_ plants (**Supplementary Table S1**). Key traits such as BM (mean 38.35 g), YPP (12.76 g), and PDW (21.02 g) exhibited positively skewed distribution (skewness = 0.42–0.52), with slightly platykurtic shapes (kurtosis = −0.26 to −0.29), suggesting a few individuals with higher values. UFG showed strong positive skewness (1.43) and leptokurtosis (2.43), reflecting a sharp peak and long tail. PH (skewness: −0.07; kurtosis: −0.29) and DFF (skewness: −0.14; kurtosis: −0.97) showed a near symmetric distribution. Traits studied at early stages, such as NDVI values at 60 DAS, showed relatively uniform distribution (skewness: −0.3; kurtosis: −1.07), suggesting consistent early canopy vigor. The Shapiro–Wilk normality test indicated that most of the traits deviated significantly from normality (*P* < 0.05), underscoring the quantitative and polygenic nature of these traits in a segregating F_2_ population.

### 3.2 Phenotypic variation associated with heterosis

**Figure 1:**
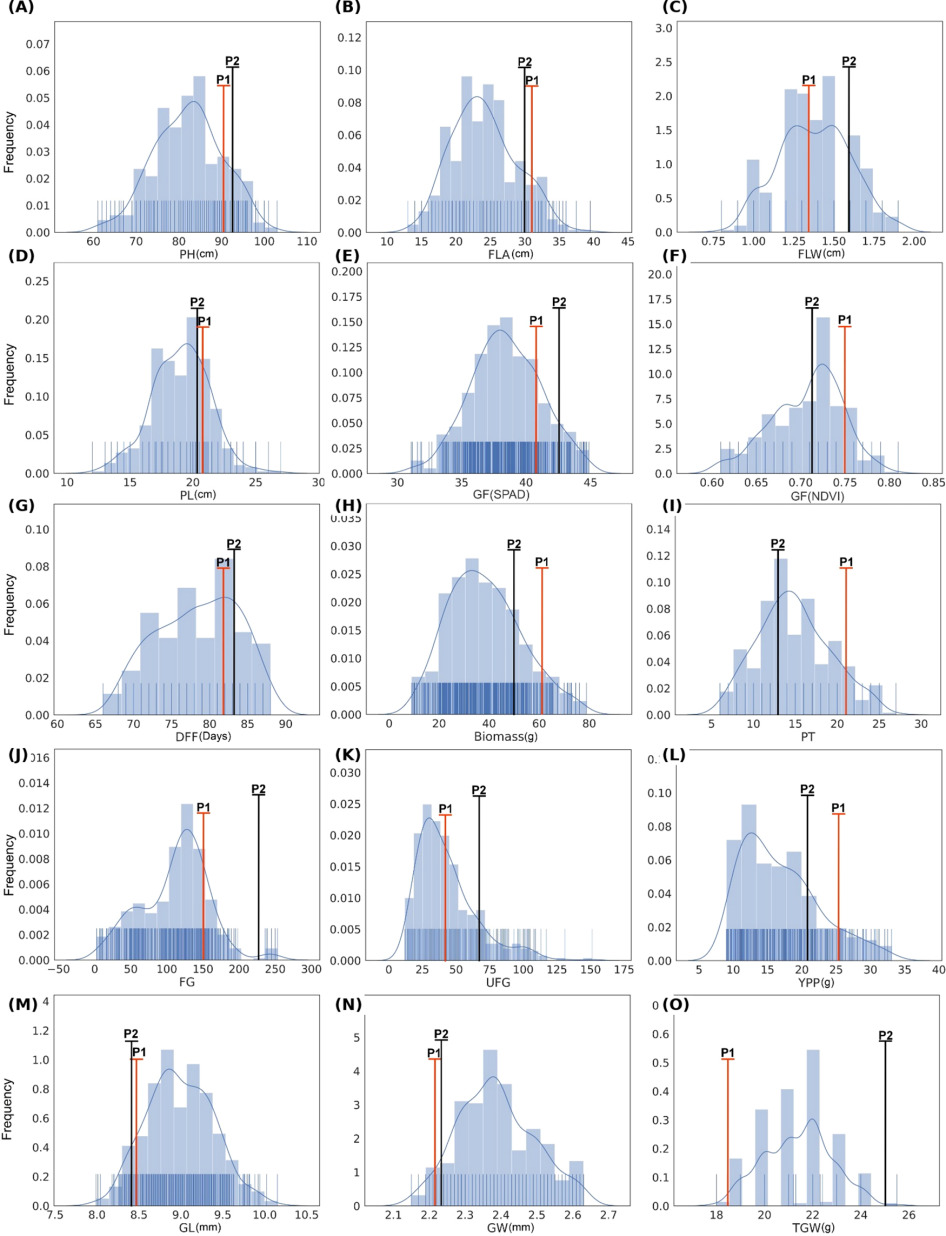
Frequency distribution of the F_2_ segregating population for physiological and Agronomic traits: **(A)** plant height (PH), **(B)** flag leaf area (FLA), **(C)** flag leaf width (FLW), **(D)** panicle length (PL), **(E)** soil plant analysis development (SPAD), **(F)** normalized difference vegetation index (NDVI), **(G)** days to 50% flowering (DFF), **(H)** biomass, **(I)** number of productive tillers (PT), **(J)** number of filled grains (FG), **(K)** number of unfilled grains (UFG), **(L)** yield per plant (YPP), **(M)** grain length (GL), **(N)** grain width (GW), and **(O)** thousand grain weight (TGW). P_1_ represents the two-line TGMS female parent and P_2_ represents the pollen parent.

The F_2_ population derived from IR144693H (IRAC43S × IRV932) exhibited extensive phenotypic variation across yield and its component traits (**Supplementary Table S1**; **Supplementary Figures 2A, B**). Transgressive segregation was observed for several traits, including PH, PL, BM, FG, UFG, YPP, and TGW, where F_2_ individuals exceeded the parent range (**Figure 1**). This suggests novel allele combinations contributing to heterosis. Mean BM was 38.35 g (variance 210.38; range 69.9 g). DFF averaged 78 days (variance 28.58; range 22 days). Mean FLL and FLW were 24.09 cm and 1.38 cm, respectively. GL and GW averaged 8.99 mm and 2.39 mm, respectively. PDW averaged 21.02 g. NDVI mean values at 30, 60, and 90 days were 0.22, 0.48, and 0.71, respectively. NT at 90 DAS averaged 14.85, and FG per panicle averaged 111.11. PH was recorded at 75.91 cm at 60 DAS and 82.14 cm at maturity. SPAD readings were 24.87, 35.10, and 38.42 at 30, 60, and 90 DAS, respectively. SCBS and SCGF averaged 264.57 and 380.06 mmol m^−2^ s^−1^, respectively. TGW averaged 21.38 g, while YPP averaged 12.76 g. Most traits exhibited slight skewness with platykurtic distributions, consistent with their quantitative inheritance.

### 3.3 Correlation and principal component analysis

**Figure 2:**
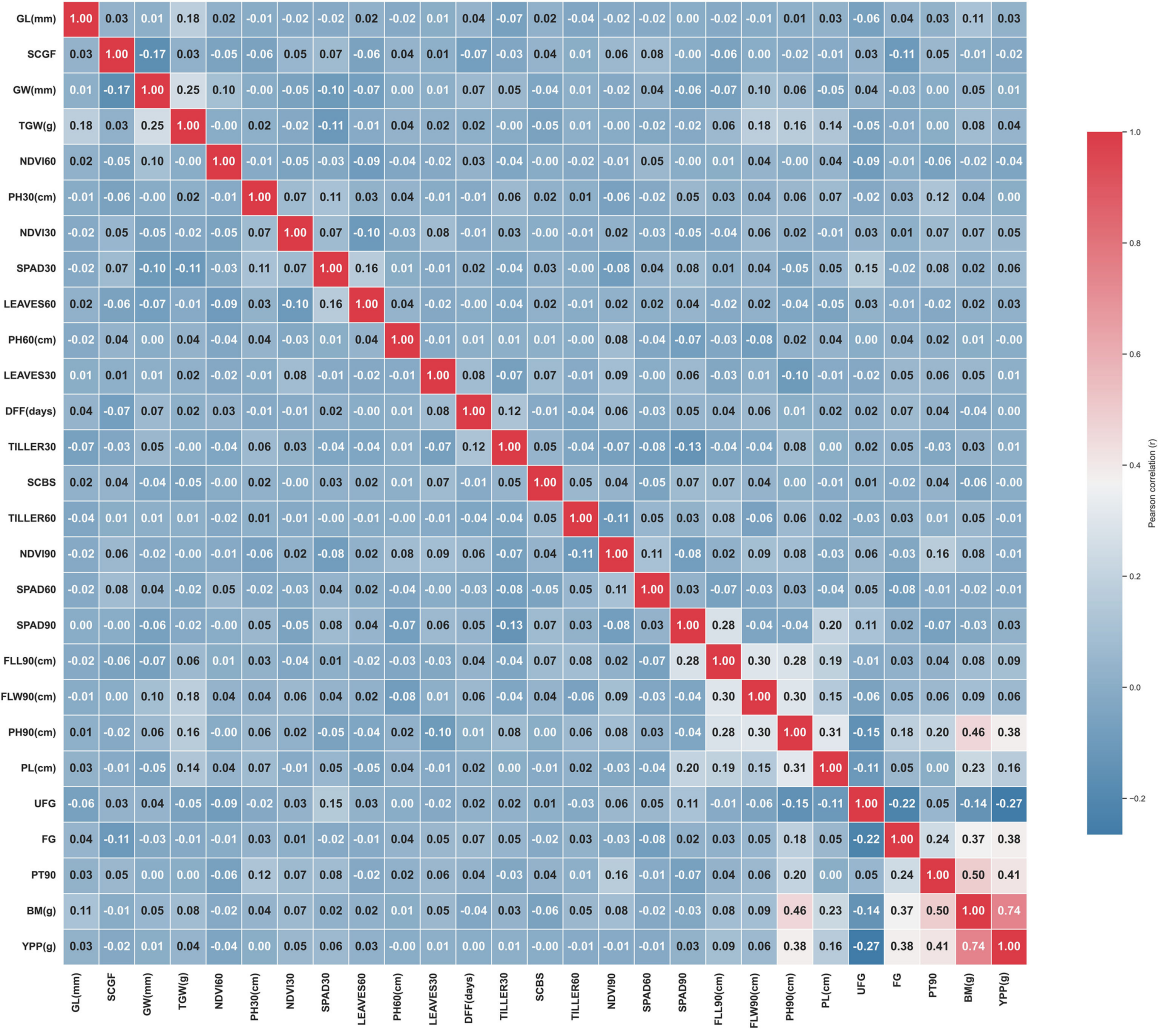
Phenotypic correlation among the yield and component traits in the F_2_ population. DTF, days to flowering; FG, filled grain; FLL, flag leaf length; FLW, flag leaf width; GL, grain length; GW, grain width; gs, stomatal conductance; TGW, thousand grain weight; NDVI, normalized difference vegetation index; PH, plant height; PL, panicle length; PT, productive tiller; SPAD, soil plant analysis development; YPP, individual plant yield.

Pearson’s correlation test determined the phenotypic correlation between yield and component traits. A total of 406 possible correlations were detected. The highest positive correlation was between YPP and BM (*r* = 0.74). In contrast, the most negative correlations were observed between UFG and YPP (*r* = −0.27) (**Figure 2**). In the PCA, positively correlated variables were grouped, while negatively correlated variables were placed on the opposite side of the origin. The distance between variables and the origin revealed the quality of the variables on the factor map. Color dots represented different F_2_ individual plants, and their position corresponded to specific trait loadings concerning PC1 and PC2 (**Supplementary Figure 3A**). The first two principal components (PC1 and PC2) explained nearly 15.9% of the total variability.

### 3.4 Construction of the linkage map

**Figure 3:**
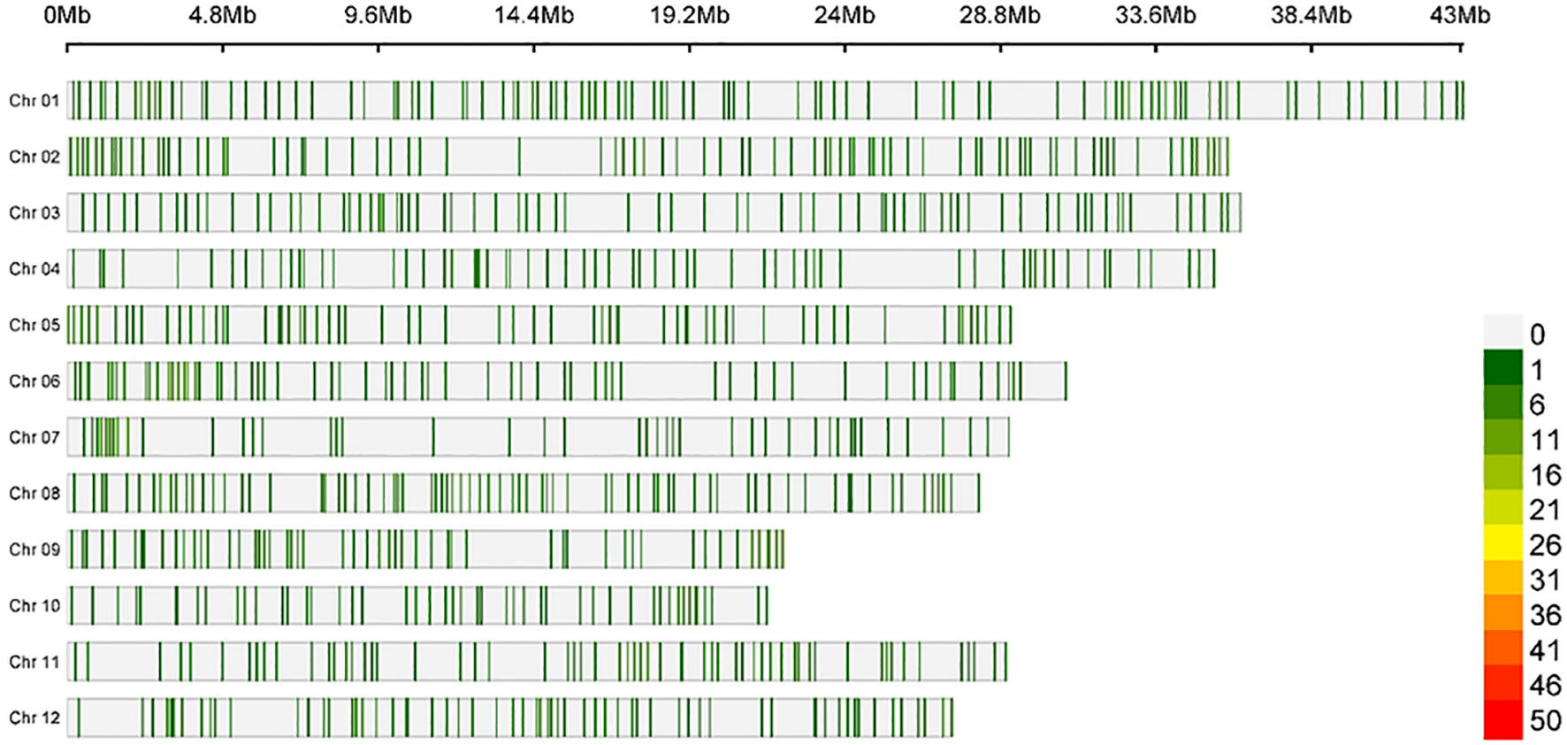
Chromosome-wise SNP density plot representing 1k-RiCA panel SNPs used in QTL analysis.

Using the 1k-RiCA SNP genotyping platform, a total of 782 high-quality polymorphic SNPs were identified and mapped across the 12 rice chromosomes. Although the SNP data were provided initially in physical positions, the markers were ordered by recombination frequencies to construct a high-resolution genetic linkage map (**Figure 3**). This dense marker distribution provided sufficient recombination-based resolution for detecting QTLs with high confidence. Once QTLs were identified on the genetic linkage map, the flanking SNP markers defining each QTL peak were aligned back to the physical reference genome using their known chromosomal positions. This alignment precisely anchored each QTL to the genome and represented it as a well-defined physical interval, facilitating downstream candidate gene identification and functional interpretation. The final genetic map spanned 367.50 cM with an average marker interval of 0.47 cM. The mapped loci among chromosomes varied from 43 (Chr 07) to 94 (Chr 01), and the map length ranged from 21.6 (Chr 10) to 43.07 cM (Chr 01). The average marker distance ranged from 0.39 (Chr 08) to 0.68 cM (Chr 07), and the lowest and highest marker densities were recorded for Chr 07 (1.48 SNPs/cM) and Chr 08 (2.60 SNPs/cM) (**Table 1**).

**Table 1:** Summary of the genetic map constructed for the F_2_ population using the 1k-RiCA panel.

Chromosome	Mapped markers	Map distance (cM)	Average marker distance	Map density(cM/locus)
Chr 01	94	43.07	0.46	2.18
Chr 02	81	35.82	0.44	2.26
Chr 03	73	36.21	0.5	2.02
Chr 04	64	35.4	0.55	1.81
Chr 05	62	29.1	0.47	2.13
Chr 06	66	30.81	0.47	2.14
Chr 07	43	29.06	0.68	1.48
Chr 08	73	28.13	0.39	2.6
Chr 09	54	22.08	0.41	2.45
Chr 10	48	21.6	0.45	2.22
Chr 11	62	28.97	0.47	2.14
Chr 12	62	27.3	0.44	2.27
Total	782	367.5	0.47	2.12

### 3.5 QTL linked with heterosis

**Figure 4:**
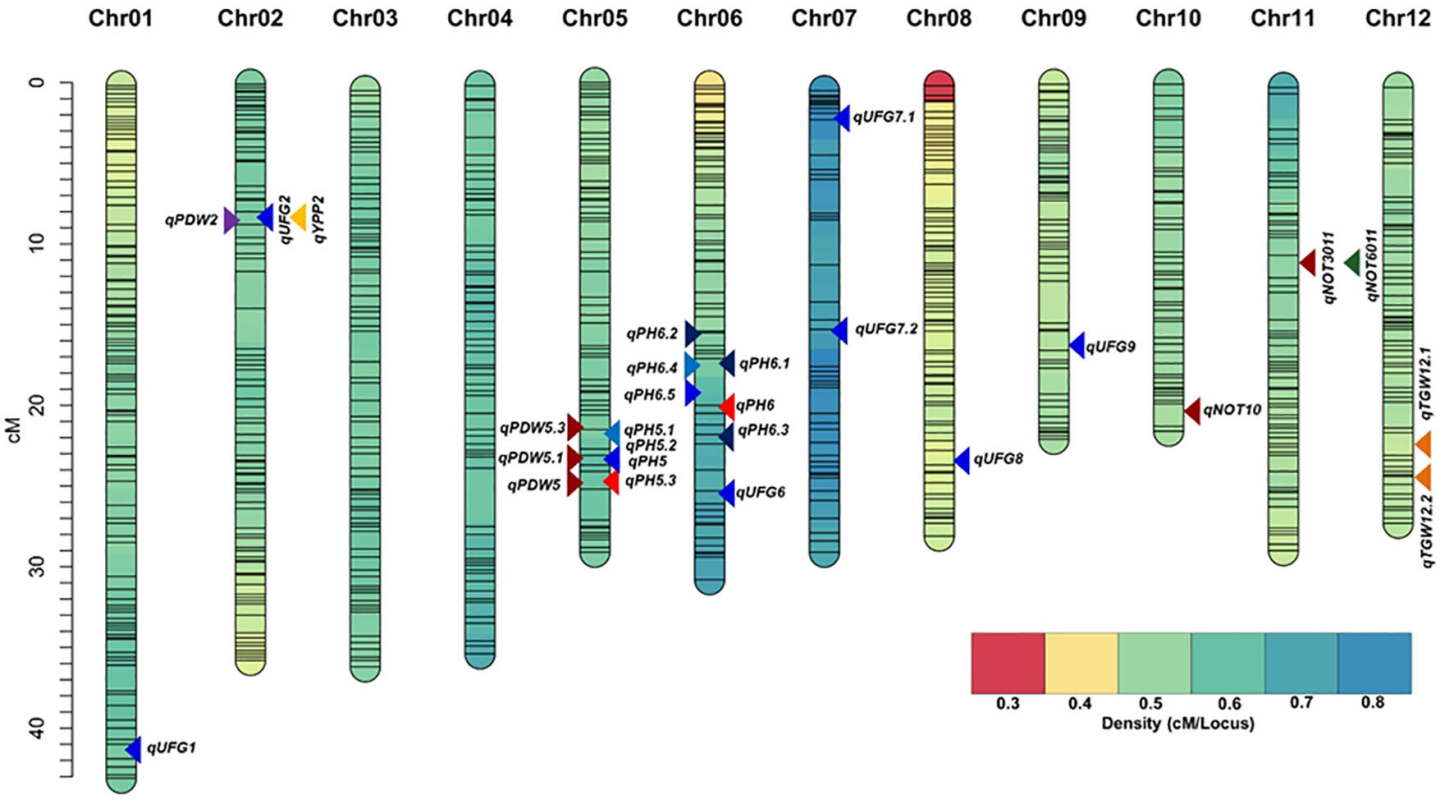
Graphical representation of the genetic map comprising 782 SNP markers for the F_2_ segregating population. Main-effect QTLs for five traits are depicted using different color symbols.

**Figure 5:**
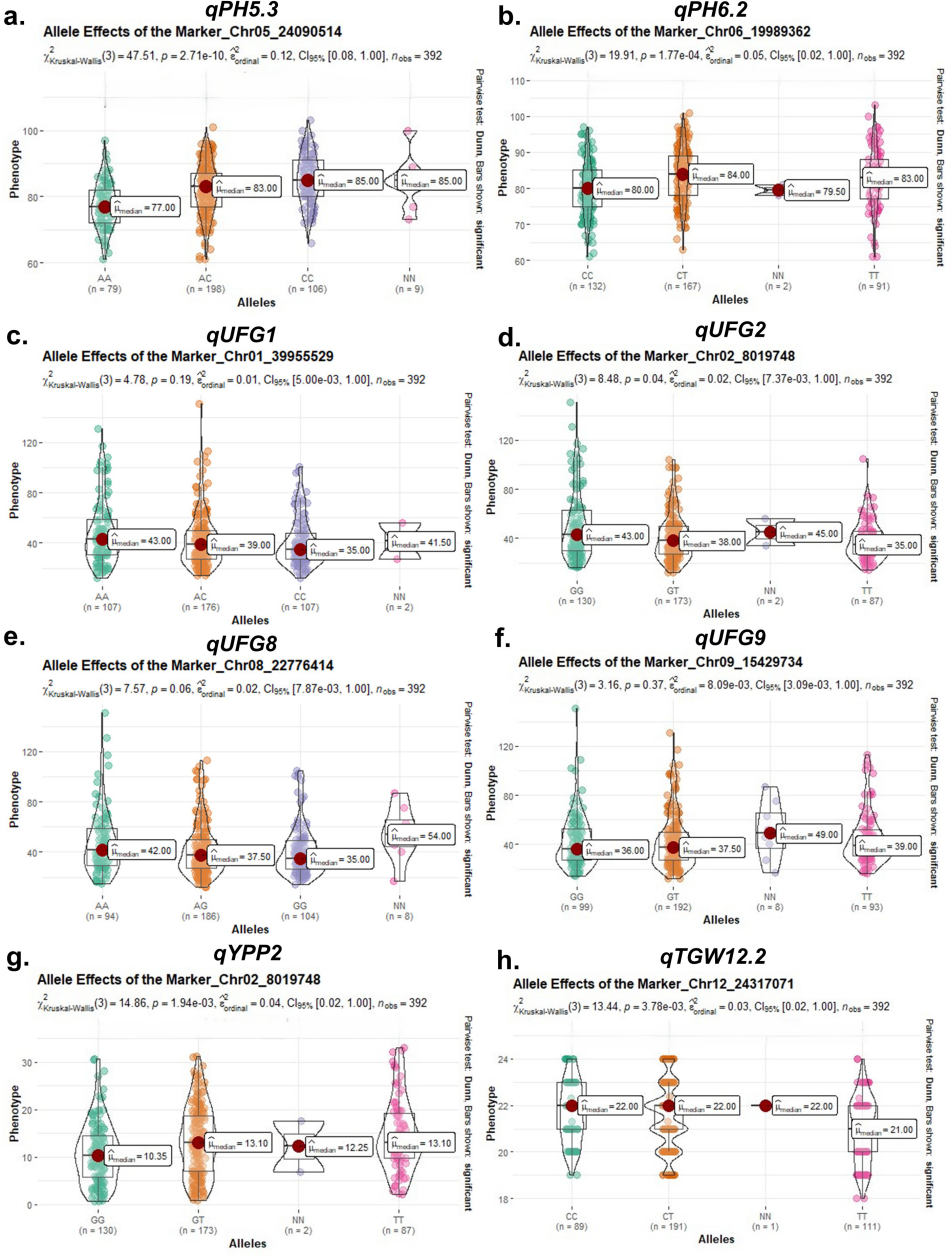
Allelic effects of major QTL-associated markers on yield and component traits in the F₂ segregating population. Violin plots show phenotypic distributions across different genotypic classes for **(a)** qPH5.3, **(b)** qPH6.2, **(c)** qUFG1, **(d)** qUFG2, **(e)** qUFG8, **(f)** qUFG9, **(g)** qYPP2, and **(h)** qTGW12.2. Central lines represent medians, and statistical significance among allelic classes was evaluated using the Kruskal-Wallis test.

Twenty-four main-effect QTLs were identified for seven traits, accounting for 4.1–67.5 of the phenotypic variances, with LOD scores ranging from 3.48 to 10.53 (**Table 2**, **Figure 4**). For UFG, seven QTLs were detected on six chromosomes (Chr 01, 02, 06, 07, 08, and 09). QTLs qUFG7 and qUFG9.1 were the major loci explaining up to 30% phenotypic variance (LOD 3.75–4.44). QTL qUFG1 was identified on chromosome 1, explaining 19.88% PVE (LOD 4.83). QTL qUFG2 on Chr 02 explained 4.14% PVE (LOD 3.52), and QTL qUFG6 on Chr 06 explained 23% PVE (LOD 4.35). Two QTLs on Chr 07 (qUFG7, qUFG7.1) explained 25.55%–30% PVE (LOD 3.75–4.44). Furthermore, qUFG8 explained 25% PVE (LOD 4.86), and QTL qUFG9.1 explained 24.49% PVE (LOD 4.44). Three QTLs for YPP and TGW (qYPP2, qTGW12.1, and qTGW12.2) were detected on Chr 02 and Chr 12, explaining 4.09%, 4.10%, and 12.04% PVE (LOD 3.48, 3.53, and 3.37), respectively. Three QTLs for NT (qNT10, qNT3011, and qNT6011) were identified on Chr 10 and 11, explaining 8.22%–67.37% PVE (LOD 3.68, 6.37, and 4.89), respectively. Interestingly, two QTLs (qNT3011 and qNT6011) were detected at both the vegetative and reproductive stages, flanked by the same markers (SNP0847_CHR11_10741559 and SNP0850_CHR11_12142966) at similar chromosome positions, indicating consistent genetic control of these traits across these developmental stages. Two QTLs for PH (qPH5 and qPH6) were detected on Chr 05 and 06, accounting for 5.03%–11.84% PVE (LOD 4.23–10.53). The allele segregation of the main-effect QTLs linked to the nearest markers on phenotype for each trait was evaluated using the Kruskal–Wallis test. The pairwise comparison test demonstrated that the differences among alleles were statistically significant in 24 main-effect QTLs for five traits (**Figure 5**). Marker segregation conformed to the expected F_2_ ratios.

**Table 2:** Summary of main-effect QTLs detected for yield and component traits using the genetic map for the F_2_ population derived from IR144693H.

Trait name	QTL Name	Chromosome	Position	Left marker	Right marker	LOD	PVE (%)	Add	Dom	Favorable allele
Plant height	qPH5	Chr 05	24.04	SNP0454_CHR05_23661597	SNP0456_CHR05_24090514	10.53	11.84	3.96	0.84	IRV932
	qPH5.1	Chr 05	23.05	SNP0452_CHR05_22711811	SNP0453_CHR05_23143060	6.45	7.35	3.09	-0.54	IRV932
	qPH5.2	Chr 05	22.05	SNP0449_CHR05_21494622	SNP0452_CHR05_22711811	6.52	8.14	3.25	-0.32	IRV932
	qPH5.3	Chr 05	25.05	SNP0456_CHR05_24090514	SNP0457_CHR05_25234430	8.32	9.91	3.62	1.44	IRV932
	qPH6	Chr 06	19.24	SNP0525_CHR06_17076390	SNP0528_CHR06_19989362	4.23	5.03	-1.52	2.52	IRAC43S
	qPH6.1	Chr 06	16.25	SNP0521_CHR06_15538142	SNP0522_CHR06_16304717	3.72	4.27	-1.48	2.23	IRAC43S
	qPH6.2	Chr 06	20.25	SNP0528_CHR06_19989362	SNP0529_CHR06_20462203	3.82	4.58	-1.4	2.43	IRAC43S
Unfilled grain	qUFG1	Chr 01	40.19	SNP0108_CHR01_39955529	SNP0109_CHR01_40699488	4.83	19.88	14.59	-11.79	IRAC43S
	qUFG2	Chr 02	8.1	SNP0146_CHR02_8019748	SNP0147_CHR02_8814718	3.52	4.14	5.43	-3.24	IRAC43S
	qUFG6	Chr 06	25.24	SNP0534_CHR06_24003856	SNP0539_CHR06_25277863	4.35	23	-53.57	-6.86	IRV932
	qUFG7.1	Chr 07	2.51	SNP0568_CHR07_2331583	SNP0570_CHR07_4481788	3.75	25.55	-5.03	40.76	IRV932
	qUFG7.2	Chr 07	15.51	SNP0581_CHR07_15340454	SNP0583_CHR07_17654305	4.44	30.05	-0.14	43.17	IRV932
	qUFG8	Chr 08	23.21	SNP0684_CHR08_22776414	SNP0685_CHR08_23713744	4.86	25.27	-26.55	-27.99	IRV932
	qUFG9	Chr 09	16.15	SNP0749_CHR09_15429734	SNP0750_CHR09_16616509	4.44	24.49	-27.5	-31.13	IRV932
Yield	qYPP2	Chr 02	8.1	SNP0146_CHR02_8019748	SNP0147_CHR02_8814718	3.48	4.09	-1.87	0.7	IRAC43S
Thousand grain weight	qTGW12.1	Chr 12	22.35	SNP0969_CHR12_21731719	SNP0974_CHR12_23066809	3.77	12.04	-0.3	0.69	IRAC43S
	qTGW12.2	Chr 12	24.35	SNP0981_CHR12_24317071	SNP0982_CHR12_24417433	3.53	4.1	-0.37	0.12	IRAC43S
Number of tillers at 30 days	qNT10	Chr 10	20.15	SNP0816_CHR10_19899160	SNP0819_CHR10_21338558	3.68	8.22	-0.13	-0.28	IRAC43S
	qNT3011	Chr 11	11.26	SNP0847_CHR11_10741559	SNP0850_CHR11_12142966	6.37	67.52	0.09	0.9	IRV932
Number of tillers at 60 days	qNT6011	Chr 11	11.26	SNP0847_CHR11_10741559	SNP0850_CHR11_12142966	4.89	44.54	0.2	4.81	IRV932
Panicle dry weight	qPDW2	Chr 02	8.1	SNP0146_CHR02_8019748	SNP0147_CHR02_8814718	4.71	5.55	-2.45	2.16	IRAC43S
Leaf dry weight	qLDW5	Chr 05	25.04	SNP0456_CHR05_24090514	SNP0457_CHR05_25234430	3.58	4.46	1.34	-0.09	IRV932
	qLDW5.1	Chr 05	23.05	SNP0452_CHR05_22711811	SNP0453_CHR05_23143060	3.64	4.21	1.25	-0.49	IRV932
	qLDW5.2	Chr 05	22.05	SNP0449_CHR05_21494622	SNP0452_CHR05_22711811	3.68	4.7	1.33	-0.39	IRV932

The “−” and “+” signs in brackets indicate negative and positive additive effects for that particular QTL.

QTLs, quantitative trait loci; LOD, likelihood of odds ratio; PVE, phenotypic variation explained.

### 3.6 Putative candidate genes linked with heterosis

**Figure 6:**
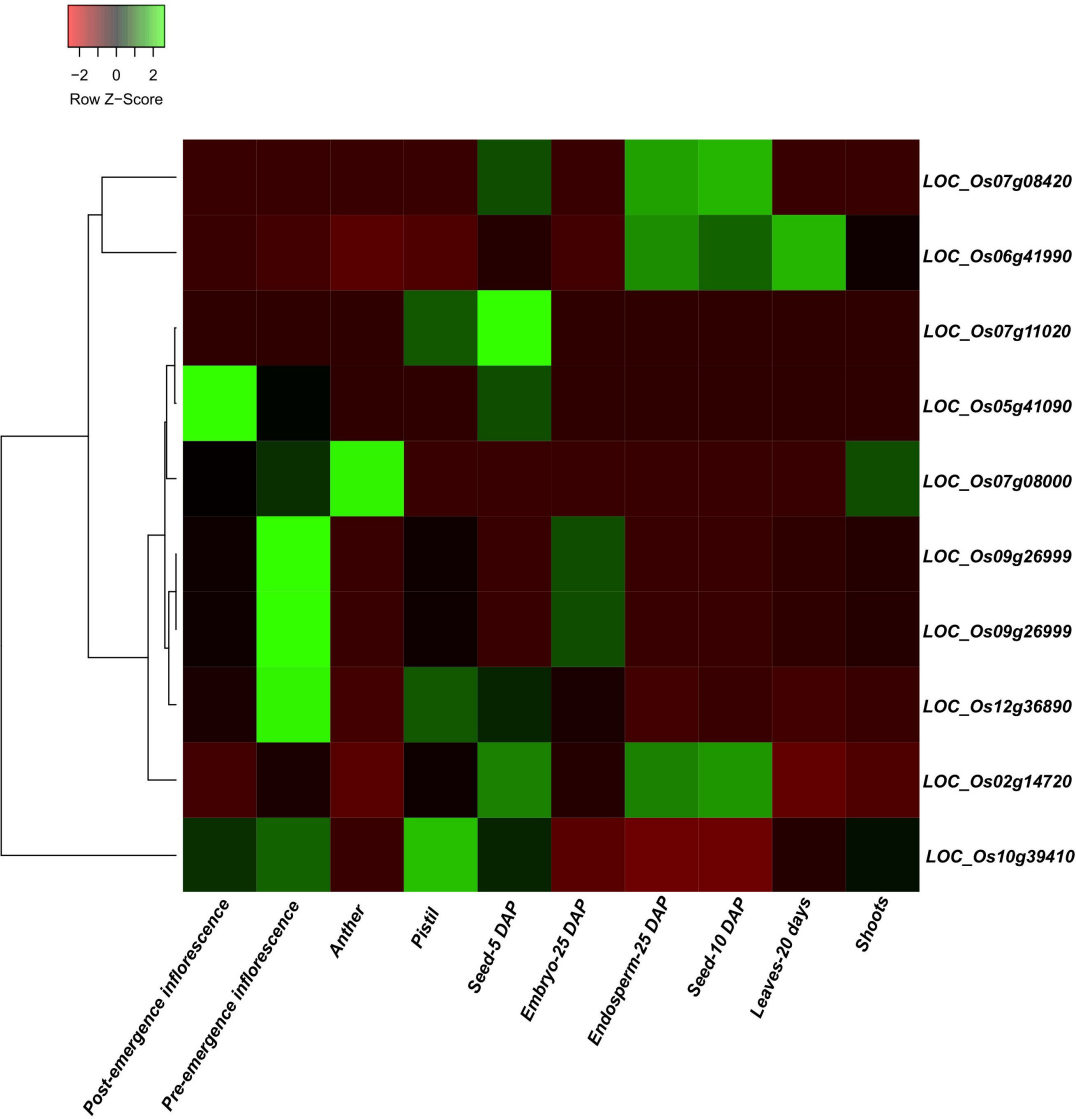
Heatmap of RNA-seq data from the RGAP database used to analyze candidate gene expression underlying main-effect QTL genomic regions. Green boxes indicate high expression levels, and red boxes indicate low expression levels among various tissues.

Across 24 detected QTL regions, 2,736 candidate gene models were identified, illustrating the extensive genomic variability underlying the examined traits. A prominent QTL hotspot was identified on Chr 02, which included qUFG2, qYPP2, and qPDW2 co-localized within a shared interval containing 90 gene models. For PH, six QTLs on chromosomes 5 and 6 (qPH5, qPH5.1, and qPH5.2 and qPH6, qPH6.1, and qPH6.2) covered 746 gene models, representing the largest gene complement among the evaluated traits. Seven QTLs associated with UFG were identified across multiple chromosomes: qUFG1 (Chr 01), qUFG2 (Chr 02), qUFG6 (Chr 06), qUFG7.1 and qUFG7.2 (Chr 07), qUFG8 (Chr 08), and qUFG9 (Chr 09). Collectively, these intervals encompassed 1,017 gene models, highlighting the polygenic nature of this trait. The single QTL for qYPP2, located on Chr 02, contained 90 gene models, while the two QTLs associated with qTGW12.1 and qTGW12.2, positioned on Chr 12, harbored 279 gene models. For NT, the QTLs qNT10 (Chr 10) and qNT3011 and qNT6011 (Chr 11) accounted for 306 genes within their respective intervals. The three QTLs associated with qLDW5, qLDW5.1, and qLDW5.2, mapped to Chr 05, collectively spanned 478 gene models, demonstrating substantial candidate gene richness in this region. Based on the candidate gene discovery approach using the Nipponbare reference genome and publicly available transcriptome expression datasets, 15 putative candidate gene loci were predicted within the confidence interval flanking markers for traits such as PH, UFG, YPP, TGW, and NT (**Table 3**). The identified locus *LOC_Os06g41990* (*SPP*) is involved in sucrose-phosphate phosphatase-regulating source-sink dynamics, and *LOC_Os02g14720* (*GW2*) participates in a ubiquitin E3 ligase that regulates grain size and weight. To investigate further the possible link between detected candidate genes and yield and component traits, we conducted expression pattern analysis of the candidate genes among various tissues using data from the Rice Genome Annotation Project database. The findings indicated that *LOC_Os06g41990* (*SPP*) was predominantly expressed in the leaf (20 days), seeds of 10 DAP (days after pollination), and endosperm of 25 DAP. The detected genes exhibited differential expression in at least one tissue across critical developmental phases (**Figure 6**).

**Table 3:** Possible candidate genes identified in the main-effect QTL genomic region associated with plant height (PH), number of unfilled grains (UFG), yield per plant (YPP), thousand grain weight (TGW), and number of tillers (NT).

Trait	QTL	Chr	Gene ID	Candidate gene	Position (bp)	Annotation
Plant height	qPH5	Chr 05	*LOC_Os05g41090*	*OsCCaMK*	23661597–24090514	Downregulation of OsCCaMK reduced plant height, number of tillers, and shoot length.
	qUFG6	Chr 06	*LOC_Os06g41990*	*SPP*	25200276–25210534	A significant quantitative trait locus, fine-mapped qSSP7, regulates the number of spikelets per panicle in rice as a single Mendelian component.
	qUFG7.1	Chr 07	*LOC_Os07g05900*	*prog1*	2839476–2839979	Inactivated prog 1 in *O. sativa* leading to erect growth, more significant grain number, and higher grain yield in cultivated rice.
			*LOC_Os07g08420*	*RISBZ1*	4322231–4326708	During the grain filling stage, RISBZI and RPBF showed interaction and compensation, which may be associated with yield and its components.
Unfilled grain			*LOC_Os07g08000*	*OsNek3*	4049754–4054523	Overexpression of OsNek3 (cytoplasmic male sterility-related protein kinase) rarely produced a peculiar structure in which the outer cell wall of the tetrad fused at the matured stage, a structure that resembles that of *Arabidopsis.*
	qUFG7.2	Chr 07	*LOC_Os07g11020*	*Rc*	6062889–6069317	Rc encodes a bHLH transcription factor that regulates red pericarp in rice.
	qUFG9	Chr 09	*LOC_Os09g26999*	*DEP1*	16411151–16415862	Variation at the DEP1 locus increases yield potential in rice.
			*LOC_Os09g26999*	*qPE9-1*	16411151–16415862	The qPE9–1 locus regulates yield and component.
Yield per plant	qYPP2	Chr 02	*LOC_Os02g14720*	*GW2*	8114961–8121925	GW2 and its function characterization proved a molecular understanding of seed development; it is a potential candidate to enhance the yield.
Thousand grain weight	qTGW12.1	Chr 12	*LOC_Os12g36890*	*dnl1*	22602880–22607315	The dnl1 mutant exhibits a thinner culm and more tillers, as well as significantly reduced grains per panicle, a lower seed setting rate, and lower grain weight compared to the wild type.
Number of tillers at 30 days	qNT10	Chr 10	*LOC_Os10g39410*	*H4*	21019255–21019903	Overexpression of OsHDAC1 in transgenic cells results in activation of the HDAC complex, which induces changes in histone acetylation *in vivo*.

## 4 Discussion

### 4.1 Genetic bases of heterosis in the TGMS-based two-line hybrid F_2_-derived population

The F_2_ population derived from IR144693H exhibited wide phenotypic variation in yield and its component traits. The presence of transgressive segregation across multiple traits confirms their polygenic and quantitative nature, which is expected when divergent parental alleles recombine to form a novel genotypic combination. Several F_2_ individuals exceeded both parents for traits such as BM, YPP, PDW, and TGW, suggesting complementary gene action. Transgressive segregation has also been commonly associated with heterosis in rice and other crops (Li et al., 2008; Yang et al., 2021). The observed deviations from normality and the strong correlation between BM and YPP (*r* = 0.74) reflect a complex underlying genetic architecture. Previous studies have demonstrated that non-additive effects, including overdominance and epistasis, play a major role in determining heterosis for yield and related traits in rice (Li et al., 2001, 2008; Dan et al., 2015). Recent genomic studies of elite rice hybrids further show that both additive and non-additive QTLs, in combination with stage-specific gene expression, contribute to hybrid vigor (Katara et al., 2020; Zhang et al., 2022). The observed transgressive segregants in F_2_ provide valuable material for breeding, as selection of individuals with extreme phenotypes can capture favorable over dominant and epistatic allele combinations. This approach is supported by evidence that targeted selection for such combinations can enhance hybrid performance and sustain heterosis in subsequent generations (Shen et al., 2014).

The Pearson correlation and PCA revealed clear trait relationships among yield and its component traits in the F_2_ population. The strong positive correlation between YPP and BM (*r* = 0.74) indicates that aboveground vegetative growth is a major determinant of final yield, consistent with the findings in other rice populations (Li et al., 2008; Yang et al., 2021). In contrast, the negative correlation between UFG and YPP (*r* = −0.27) suggests that spikelet sterility significantly constrains yield potential. PCA further supported these findings by grouping positively correlated traits together and placing negatively associated traits on the opposite side of the factor map. The first two PCs explained 15.9% of the total variability, highlighting the polygenic and complex inheritance of yield-related traits, as also observed in previous F_2_ studies in rice (Katara et al., 2020; Zhang et al., 2022). The construction of a high-resolution linkage map using 782 polymorphic SNPs provided a robust framework for identifying QTLs with high confidence. SNPs were well distributed across the 12 chromosomes, with marker density varying from 0.39 to 0.68 cM and chromosome lengths ranging from 21.6 to 43.07 cM, ensuring adequate coverage for detecting recombination events. By aligning flanking SNPs to the physical genome, each QTL was precisely anchored to defined genomic intervals, enabling downstream candidate gene identification and functional interpretation. Such dense, recombination-based maps are critical for resolving QTLs with high confidence and have been successfully applied in other rice F_2_ populations to dissect complex traits such as yield and biomass (Li et al., 2001; Shen et al., 2014). The combined use of correlation, PCA, and high-resolution linkage mapping underscores the potential of integrating phenotypic and genotypic data to understand trait architecture in segregating populations. Identification of trait correlations provides insight into indirect selection strategies, while precise QTL localization facilitates the discovery of candidate genes underlying heterosis and yield performance. This approach enhances the efficiency of breeding programs targeting complex traits in rice (Li et al., 2008; Zhang et al., 2022).

The 24 QTLs identified in this study explained 4.1% to 67.5% of phenotypic variance, emphasizing the polygenic regulation of the traits examined. Among them, loci for NT (qNT3011, qNT6011) and UFG (qUFG7.2, qUFG9) regions with consistent genetic effects are expected to underpin hybrid vigor. For instance, QTLs for UFG on chromosomes 7 and 9 correspond with panicle development genes reported by Yin et al. (2021) and Zhang et al. (2025), while qTGW12.1 aligns to regions associated with grain weight (Ding et al., 2015). QTLs for NT on chromosome 11, potentially representing novel loci not widely reported in TGMS-based two-line populations, open up new insights into the genetic regulation of vegetative vigor. Integration of candidate gene analysis with tissue-specific expression enables our study to narrow in on TGMS-based two-line hybrid and directly link QTLs with expression-supported genes. Such integration may help bridge the gap between genetic mapping and biological function, thereby strengthening the use of QTL-linked markers in hybrid rice breeding technology. These results confirm previously reported findings, showing that F_2_ populations efficiently capture additive and dominance interactions that contribute to heterosis (Huang et al., 2016; Koide et al., 2019). Notably, the QTLs detected in this study revealed both additive and dominance effects, indicating a mixed genetic basis of heterosis. In this study, qNT3011 and qNT6011, for NT, were located on chromosome 11, which were associated with up to 67% PVE, indicating a significant role of overdominance in tillering dynamics. Conversely, for PH (qPH5, qPH6) and TGW (qTGW12.1) QTLs, an additive as well as overdominance effect contribution was demonstrated. This genetic architecture supports the hypothesis that heterosis arises from a complex interplay of additive, dominance, and epistatic effects, with certain genomic regions disproportionately influencing yield potential (Liu et al., 2020; Xie et al., 2022).

### 4.2 Candidate gene insights and molecular regulation

Analysis of 24 QTLs associated with key agronomic traits in rice identified 2,736 gene models, underscoring the complex genetic control of yield and its components. A prominent QTL cluster was observed on chromosome 2, where qUFG2, qYPP2, and qPDW2 co-localized within a shared interval spanning 90 gene models, suggesting pleiotropic effects or tight linkage. Among these, *GW2* (*LOC_Os02g14720*) has been extensively characterized as a key regulator of seed development, controlling grain width and weight through ubiquitin-mediated pathways (Song et al., 2007). Its co-localization with QTLs for UFG and PDW indicated a genomic hub that likely coordinates assimilate partitioning and grain filling efficiency. Similarly, PH QTLs on chromosomes 5 and 6, including *OsCCaMK* (*LOC_Os05g41090*), demonstrate pleiotropic influence by modulating shoot length, NT, and overall plant architecture (Ikeda et al., 2011). These results align with previous studies showing that complex traits such as height are controlled by multiple loci with both additive and pleiotropic effects (Dwivedi et al., 2024; Liu et al., 2024). QTLs for UFG on chromosomes 6, 7, and 9 encompass functionally relevant candidate genes such as *SPP* (*LOC_Os06g41990*), which regulates spikelet number per panicle (Xing et al., 2008); *prog1* (*LOC_Os07g05900)*, which promotes erect growth and increased grain number in cultivated rice (Jin et al., 2008); and *RISBZ1* (*LOC_Os07g08420*), which interacts with RPBF during grain filling to regulate starch biosynthesis and grain weight (Yamamoto et al., 2006). Additionally, *Rc* (*LOC_Os07g11020*) influences pericarp pigmentation, indirectly affecting grain development (Sweeney et al., 2006), and *DEP1/qPE9-1* (*LOC_Os09g26999*) enhances panicle architecture and yield potential (Huang et al., 2009). For TGW, *dnl1* (*LOC_Os12g36890*) affects culm strength and grain weight, while *H4/OsHDAC1* (*LOC_Os10g39410*), identified under qNT10, modifies histone acetylation, impacting meristem activity and tiller initiation (Banerjee and Roychoudhury, 2018; Liu et al., 2023). Together, these findings confirm that reproductive and vegetative traits are genetically coordinated through both structural and regulatory genes, emphasizing the polygenic nature of yield-related traits.

Also, a well-known functional clue into molecular pathways was associated with yield heterosis. For example, *SPP* (*LOC_Os06g41990*) was found near qUFG6, which encodes a sucrose-phosphate phosphatase that regulates source-sink dynamics and sugar allocation to grains. The gene expression in the leaves and endosperm aligns with its contribution in enhancing grain filling and reducing the number of UFG. Recent studies have reported that carbohydrate partitioning is the central hub of heterosis (Bozdar et al., 2025). Correspondingly, *GW2* (*LOC_Os02g14720*) was identified and linked with qYPP2, which encodes an E3 ubiquitin ligase regulating grain size and weight and is expected to contribute to higher biomass accumulation (Bozdar et al., 2025), emphasizing its contribution to biomass and yield heterosis. The putative candidate gene loci determining the plant architecture, such as *OsCCaMK* (*LOC_Os05g41090*) and *PROG1* (*LOC_Os07g05900*), are linked with PH and NT, and these putative loci participate in regulating developmental plasticity and structural integrity, ensuring the optimal plant type for efficient resource utilization. Moreover, *DEP1* (*LOC_Os09g26999*) and qPE9-1, which are mapped within the qUFG9 interval region, are well-understood regulators of panicle architecture for grain number (Zhang et al., 2021; Wang et al., 2025). The existence of such functional loci within the QTL interval provides direct molecular evidence linking genotype to heterotic phenotypes. Some candidate genes suggest less obvious regulatory layers; histone *H4* (*LOC_Os10g39410*) was detected with qNT10, indicating that chromatin remodeling and cytokinin signaling may influence tiller initiation. Likewise, *dnl1* (*TGW12.1*) highlights the importance of cell wall integrity for grain weight in hybrid performance. These studies complement current research, which indicates that heterosis is not solely genetic but also mediated through transcriptomic and epigenetic remodeling (Gu et al., 2023; Zhou et al., 2024).

### 4.3 Breeding implications for two-line hybrid rice

The integration of candidate gene information with QTL intervals provides valuable insight for breeding strategies in TGMS-based two-line hybrid rice. The chromosome 2 hotspot represents a key target for MAS, as it simultaneously influences PDW, grain filling efficiency, and YPP. However, functional validation of key genes through CRISPR/Cas9, overexpression, or near-isogenic lines will clarify causal relationships and allow precise allele selection. Furthermore, epistatic interactions among QTLs for PH, NT, and yield components underscore the need to consider genomic context in breeding programs (Huang et al., 2010). Overall, these results enhance our understanding of multilocus networks controlling rice yield and provide a roadmap for exploiting favorable alleles to optimize plant architecture and productivity. QTLs and putative candidate genes have potential implications for TGMS-based two-line hybrid breeding. Key loci such as qNT3011 and qNT6011 can be targeted through MAS to improve NT. Meanwhile, qUFG6 and qUFG9 may provide entry points for reducing spikelet sterility and enhancing sink strength. Putative candidate genes like *SPP* and *GW2* display a potential target for functional validation and genome editing, enabling precise improvement of yield and its component traits. Moreover, the identification of epigenetic regulators, such as histone *H4* (*LOC_Os10g39410*), underscores the importance of incorporating epigenomic insight into molecular breeding strategies to more fully exploit heterosis. Future efforts combining QTL mapping with transcriptomic and epigenomic profiling will deepen our understanding of yield heterosis and accelerate the development of high-yielding two-line rice hybrid.

## 5 Conclusion

This study investigated the genetic bases of heterosis in a TGMS-based two-line rice hybrid-derived F_2_ segregating population. Using high-density SNP genotyping, 24 QTLs associated with major agronomic traits were identified, spanning 2,736 gene models across multiple chromosomes. Notably, a major QTL cluster on chromosome 2 co-localized QTLs for UFG, YPP, and PDW, suggesting the genomic hub regulating assimilate partitioning, grain filling, and biomass allocation. Key QTLs—such as qNT3011 and qNT6011 for NT across developmental stages and qUFG7.2 and qUFG9 for UFG—explained the variation. Grain weight variation was linked to qTGW12.1 on Chr 12. These findings emphasize the polygenic and layered nature of heterosis in hybrid rice. The identified QTLs showed mixed genetic effects, with additive and partial dominance and overdominance effects. Overdominance predominated for NT and TGW, whereas additive and partial dominance effects governed most of the other traits, and such a pattern reflects the combined action of complementary and non-additive cross-talk contributing to heterosis. In addition, putative candidate loci provide supportive evidence linking structural and regulatory genes to QTL effects. Genes such as *SPP*, *GW2*, *DEP1*, and *OsCCaMK* showed strong coordination with key traits ranging from carbohydrate partitioning to panicle structure and PH regulation. Genes including histone *H4* and *dnl1* suggested the importance of chromatin remodeling and cell wall integrity in modulating hybrid performance. These findings highlight genomic regions that plant breeders can utilize for direct use in MAS in TGMS-based hybrid rice breeding. Ultimately, this study reinforces that heterosis arises from the combined action of structural, regulatory, and epigenetic factors. Further integration of QTL mapping with transcriptomic and epigenomic studies will bridge the gap between genotype and phenotype, accelerating the development of high-yielding two-line rice hybrids.

## Data Availability

The datasets presented in this study can be found in online repositories. The names of the repository/repositories and accession number(s) can be found in the article/**Supplementary Material**.

## Supplementary material

Supplementary Table 1 Summary of Phenotypic Statistics for All Agronomic and Physiological Traits.

Supplementary Table 2 Candidate Gene Expression Overlapping QTL Region.

Supplementary Table 3 List of Putative Candidates Genes Identified Within QTL Region.
