# Supplementary figures and images for: Unraveling the genetic and molecular bases of heterosis in a TGMS-based two-line rice hybrid derived F2 segregating population
Source: Front Plant Sci. 2026 Jan 16;16:1722476. doi: 10.3389/fpls.2025.1722476 (PMC12867038; doi:10.3389/fpls.2025.1722476)

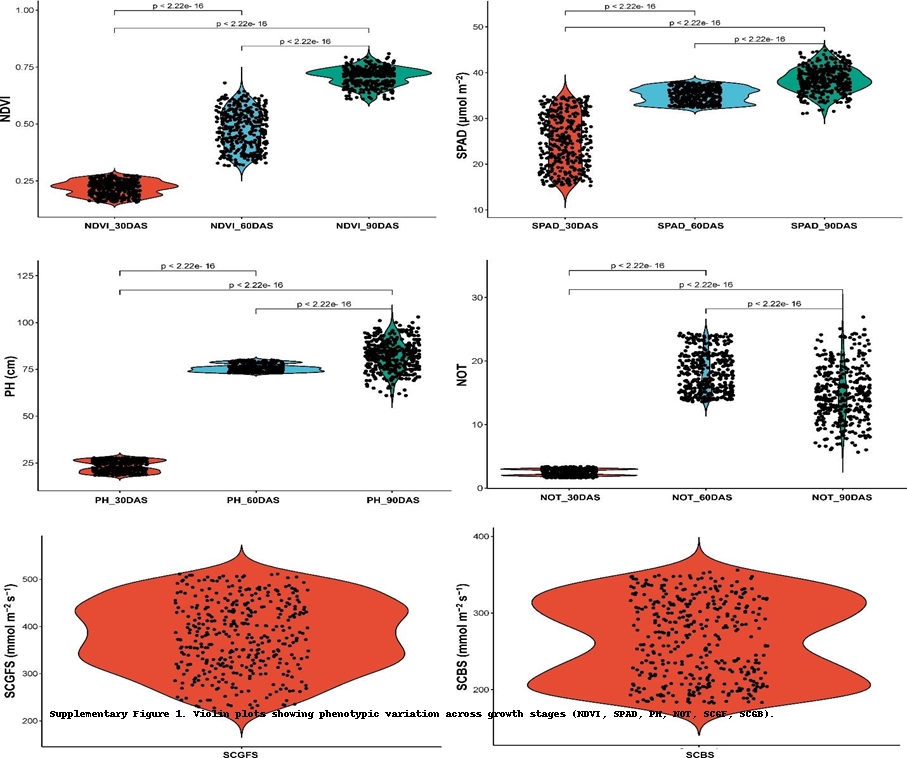

Supplement: Supplementary file 4 [file Image1.jpeg]

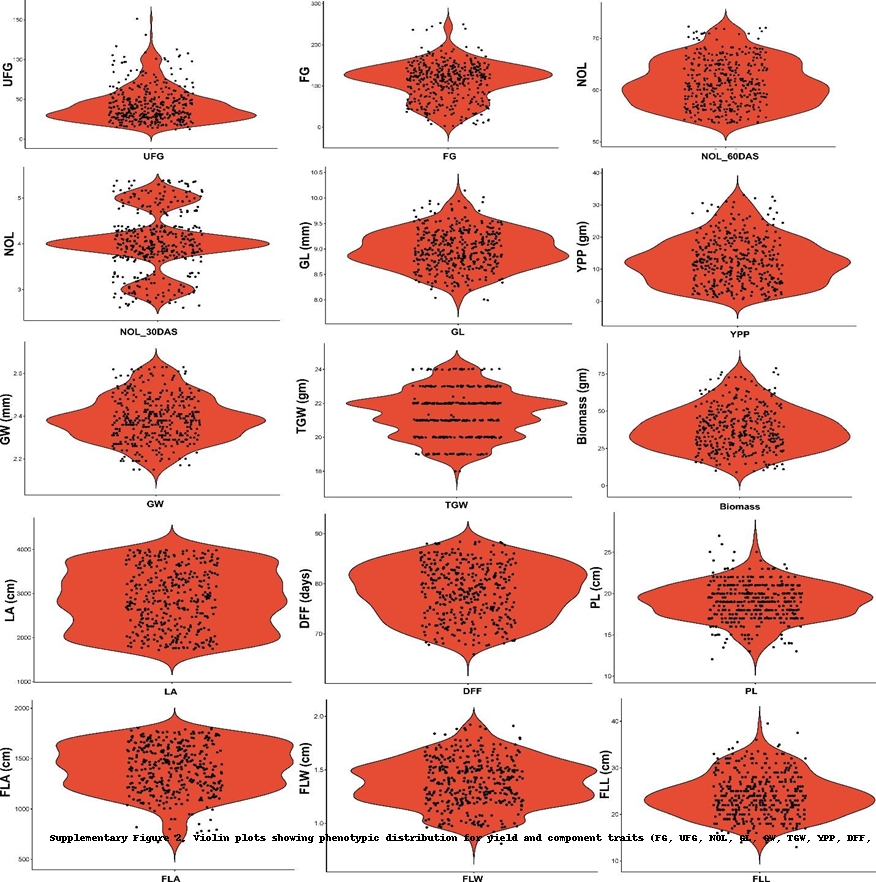

Supplement: Supplementary file 5 [file Image2.jpeg]

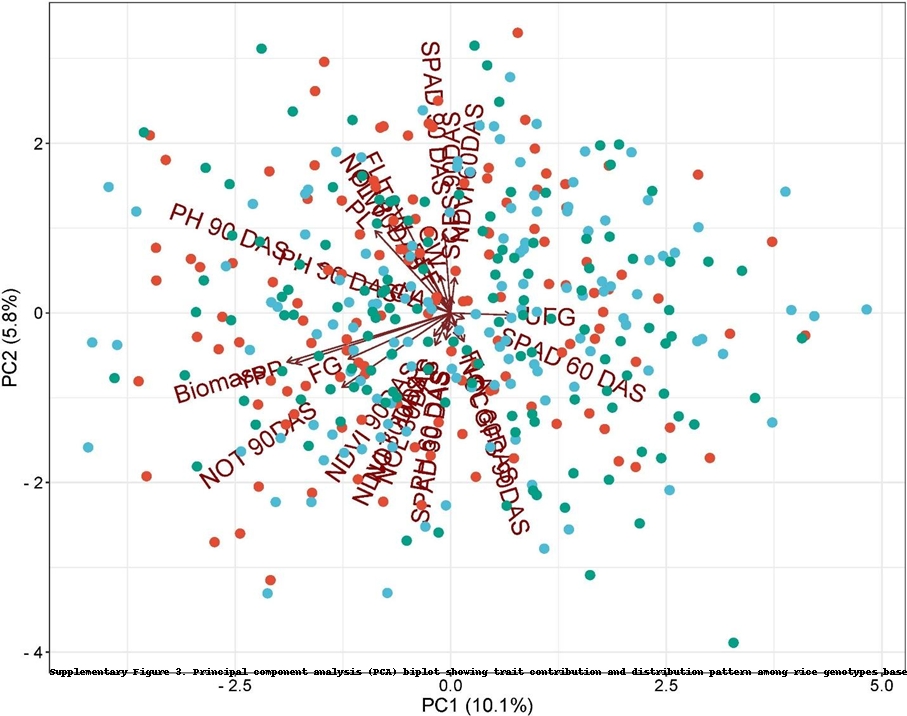

Supplement: Supplementary file 6 [file Image3.jpeg]
